# Genetic Risk Factors for Poor Cognitive Outcome Following Brain Insult—A Systematic Review

**DOI:** 10.1002/brb3.71173

**Published:** 2025-12-31

**Authors:** Tora Dunås, Sophia Leiss, Alba Corell, Thomas Skoglund, Anna Dénes, Helena Carén, Anja Smits, Isabelle Rydén, Asgeir Jakola

**Affiliations:** ^1^ Department of Clinical Neuroscience, Institute of Neuroscience and Physiology Sahlgrenska Academy, University of Gothenburg Gothenburg Sweden; ^2^ Department of Neurosurgery Sahlgrenska University Hospital Gothenburg Sweden; ^3^ Sahlgrenska Centre for Cancer Research, Department of Medical Biochemistry and Cell Biology, Institute of Biomedicine Sahlgrenska Academy, University of Gothenburg Gothenburg Sweden; ^4^ Department of Medical Sciences, Neurology Uppsala University Uppsala Sweden; ^5^ Department of Neurology Sahlgrenska University Hospital Gothenburg Sweden

**Keywords:** brain insult, cognition, genetics

## Abstract

**Introduction:**

Cognitive outcomes following brain insult are shaped by a range of factors, including genetic predispositions. Emerging evidence indicates that specific genetic variants may affect the susceptibility to cognitive impairment in individual patients. In this systematic review we summarize the evidence for genetic variants on cognitive outcomes following brain insults.

**Methods:**

A systematic search was conducted in PubMed, Embase, PsycINFO, bioRxiv, medRxiv, reference lists, and ClinicalTrials.gov to identify studies published before June 14, 2023, reporting associations between genetic variants and cognitive outcomes following brain insults. Only studies conducted in humans and published in English were included. A broad definition of brain insults was applied, with a primary focus on stroke, traumatic brain injury (TBI), and brain tumors. All articles underwent bias assessment using the JBI critical appraisal tools.

**Results:**

Of the 121 studies included, 80 (66%) were rated as low risk of bias. The *APOE* gene was investigated in 56% of TBI studies, 52% of stroke studies, and 43% of studies on other brain injuries. Of the 74 studies on *APOE*, 50 (68%) focused on the *ε*4 allele, with 39 studies (87%) reporting associations between the *ε*4 allele and worse cognitive outcomes. The *BDNF* rs6265 polymorphism was examined in 18 studies, 15 of which reported significant effects on cognitive outcomes. However, the direction of these effects was inconsistent, with seven studies linking the G allele and seven the A allele to worse cognitive outcomes. For the *COMT* rs4680 polymorphism, nine out of 12 studies reported worsened cognitive outcomes linked to the G allele, while several reported a protective association for the A allele. Injury‐ and population‐specific patterns were not consistent.

**Conclusion:**

This systematic review suggests that *APOE‐*ε4 and potentially the G allele of *COMT* rs4680 are associated with poor cognitive outcomes following brain insults. The type of brain injury does not appear to influence whether genetic variants predispose to favorable or unfavorable cognitive outcomes. Future research may benefit from focusing on these markers, particularly in larger datasets, to validate these findings.

## Graphical abstract

This systematic review summarizes genetic predictors of cognitive outcomes following stroke, traumatic brain injury, and other brain insults. *APOE‐*ε4 consistently predicts poorer cognitive recovery. Evidence for the *COMT* rs4680 G allele suggests adverse associations, while *BDNF* findings remain inconsistent.

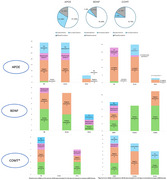

## 1 Introduction

Cognitive impairments, referring to difficulties in mental functions such as learning, thinking, reasoning, decision‐making and problem‐solving, are frequently observed following various brain injuries, such as brain tumors. These symptoms pose a significant risk of negatively impacting activities of daily life in addition to affecting the quality of life (Frances et al. 2022). However, in clinical practice we observe a large heterogeneity in outcome that cannot be explained solely by different brain locations and different types of injuries (Kirkman et al. 2022). This raises the question of whether intrinsic host factors, such as genetic susceptibility, may play a role in moderating these outcomes. Thus, exploring genetic variations in relation to recovery from various brain insults can offer valuable insights.

Genetic polymorphisms have been explored in various conditions, with several genetic risk factors identified for their role in cognitive outcomes after brain insults (Kurowski et al. 2019; Lakshmipathy et al. 2024). Clinically, the main challenge lies in identifying reliable markers for diagnostics and prognostication. For patients with brain tumors, understanding genetic susceptibility to cognitive deterioration could be particularly relevant when providing neurotoxic treatments such as surgery and radiotherapy (Correa 2008). In such cases, knowledge of susceptibility for poor recovery could alter decisions on both *if* and *when* to provide treatment, especially in patients with more favorable tumor types and longer life expectancies (Verma et al. 2020).

Existing reviews are typically diagnosis‐ or gene‐specific, leaving no cross‐acquired brain injury synthesis. This systematic review integrates evidence across TBI, stroke, and brain tumors, organizing findings by gene and injury context, and weighting interpretation by JBI risk of bias. The aim is to identify where genetic effects on cognition are consistent, explain sources of divergence, and offer practical recommendations to improve comparability and clinical utility, providing a foundation for integrating genetic markers into clinical practice to personalize care after brain injury.

## 2 Materials and Methods

The protocol was registered in PROSPERO with the ID CRD42023432679.

Studies were eligible for inclusion if they investigated how genotype affected cognition in humans that had suffered an acquired brain injury. Only reports in English were included.

We searched PubMed, Embase, and PsycINFO, as well as the preprint repositories bioRxiv and medRxiv, from database inception to June 14, 2023 (last search date). No publication date limits were applied. We also scanned the reference lists of identified studies and looked for ongoing studies identified via ClinicalTrials.gov. Records not published as full texts, such as conference abstracts, were excluded.

The search prompt had three parts and was a combination of MeSH terms and title/abstract words:

1. Genes, genetics, polymorphism, or *APOE*
2. Cognition, cogni*, or neuropsychologic*
3. Stroke, brain neoplasm, brain injuries, intracranial hemorrhages, glioma, brain surgery, brain tumoy*, brain hemorrhage*, brain injury*, brain cancer*, or neural injury*.

*APOE* was included in the search strategy to capture studies mentioning it as a risk factor, even when genetics is not explicitly referenced in the title or abstract. The full search prompt used is provided in Supplementary Material 1.

**FIGURE 1:**
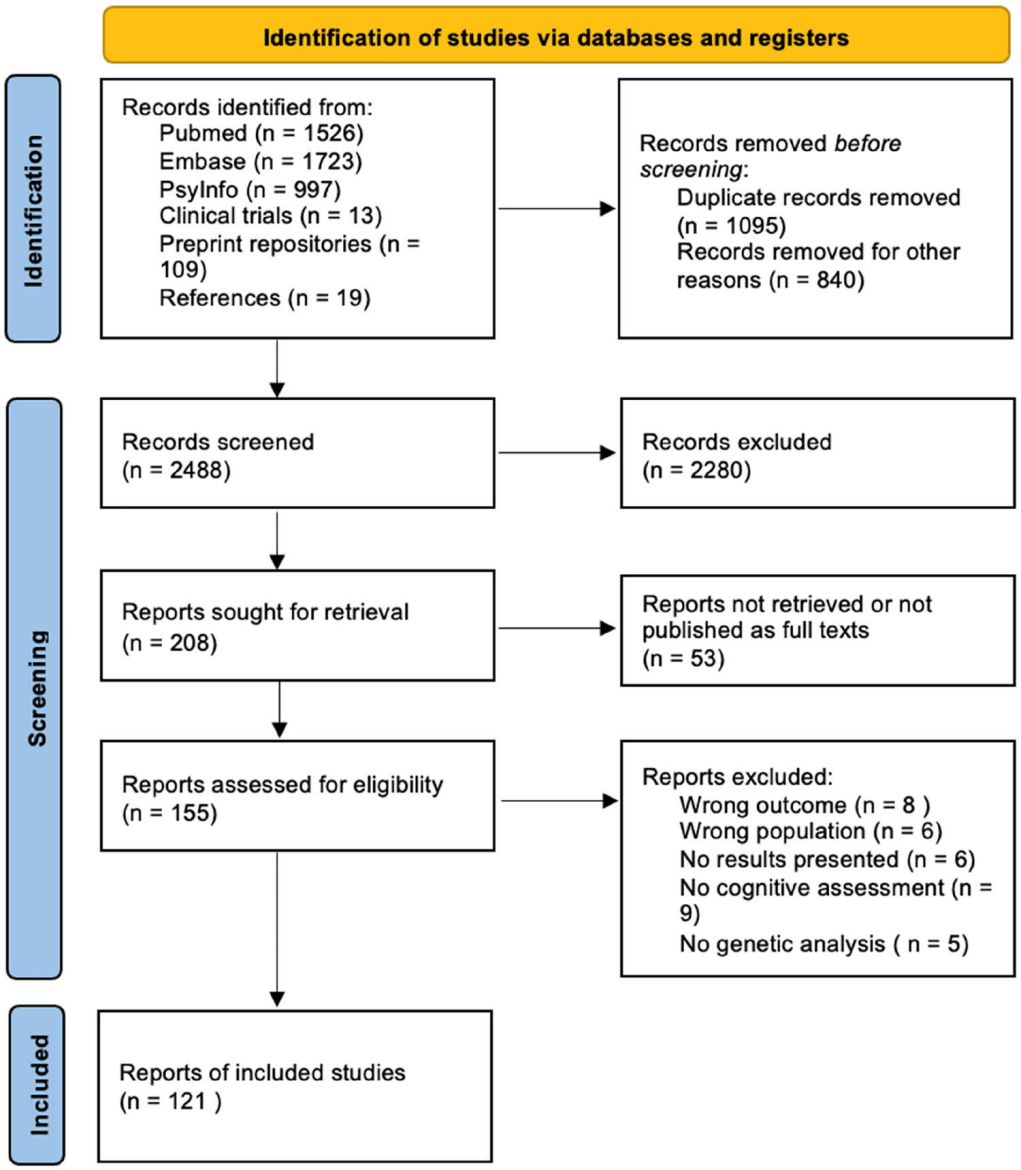
PRISMA flowchart of information about article selection.

After initial searches, the review tool *Rayyan* (Ouzzani et al. 2016) was used to screen articles for inclusion/exclusion criteria. We followed PRISMA 2020 and document identification and selection in a PRISMA flow diagram. Counts for each stage are shown in Figure 1.

Screening was conducted in two stages (title/abstract, then full text) against prespecified eligibility criteria. Each record was screened independently by two reviewers. Conflicts were first resolved by discussion; unresolved disagreements were adjudicated by a third

Reviewer (A. D.), and when needed T. D. acted as tie‐breaker. Reasons for exclusion at full‐text screening were recorded.

Data extraction was performed by one reviewer (S. L.), using a piloted Excel form created by T. D., and verified by a second reviewer (T. D.). Besides bibliographic information, data was extracted on aim, population, population size, recruitment, time span, outcome, investigated genes, type of cognitive assessment, main results, and conclusions. Discrepancies were resolved by consensus or, if needed, adjudication by the third reviewer (A. D.); any remaining issues were escalated to T. D.

The risk of bias was assessed using the JBI critical appraisal tools; checklists were selected based on study type.

A structured narrative synthesis was utilized. For each included study we tabulated design, population, brain‐injury context (stroke, TBI, brain tumor), cognitive outcomes, genotypes, and main findings. We then qualitatively summarized patterns across studies, focusing on the direction and consistency of gene–cognition associations within and across injury contexts. When multiple cognitive measures were reported, we described the most used global measures and key domains as presented by the authors. Risk‐of‐bias assessments (JBI) informed interpretation; studies at higher risk of bias were down‐weighted narratively and did not drive conclusions.

## 3 Results

The database searches resulted in 4404 records; an additional 19 publications were identified from references. After the inclusion process outlined in Figure 1, 121 articles were included for analysis.

Of the 121 studies, 80 (66%) were assessed as having a low risk of bias, 26 (22%) as having a moderate risk of bias, and 15 (12%) as having a higher risk of bias. We placed significant emphasis on sample size as a critical factor in our bias assessment, particularly when the sample size was below 50 participants (*n* = 17). While longitudinal studies generally have a lower risk of bias, data regarding follow‐up, drop‐out reasoning, and potential impacts on outcomes were often not reported. Among the 80 studies assessed as having a low risk of bias, we identified 51 longitudinal studies, 17 cross‐sectional studies, and 12 case‐control studies (Supplementary Table 2).

### 3.1 Study Characteristics

This systematic review includes 80/121 (66%) longitudinal studies, 23/121 (19%) cross‐sectional studies and 18/121 (15%) case‐control studies. Out of the 80 longitudinal studies, 31/80 (39%) had a follow‐up period of ≤6 months.

Of the 121 included studies, 77 (64%) had baseline measurements within 6 months. Our review included international data, mainly from the United States of America (43%), followed by China (9%), Australia (7%), or the United Kingdom (6%).

Among the 121 included articles, 79% focused on adult populations, with 12% specifically targeting elderly individuals, defined by a mean age of >70 years. A smaller proportion of the studies (5%) examined pediatric populations.

The studies represented traumatic brain injuries (TBI) (*n* = 56, 46%), stroke or cerebrovascular events (n = 45, 37%), and other injuries (*n* = 20, 17%), such as brain tumors (*n* = 17)—six of the 17 brain tumor studies were in children.

**FIGURE 2:**
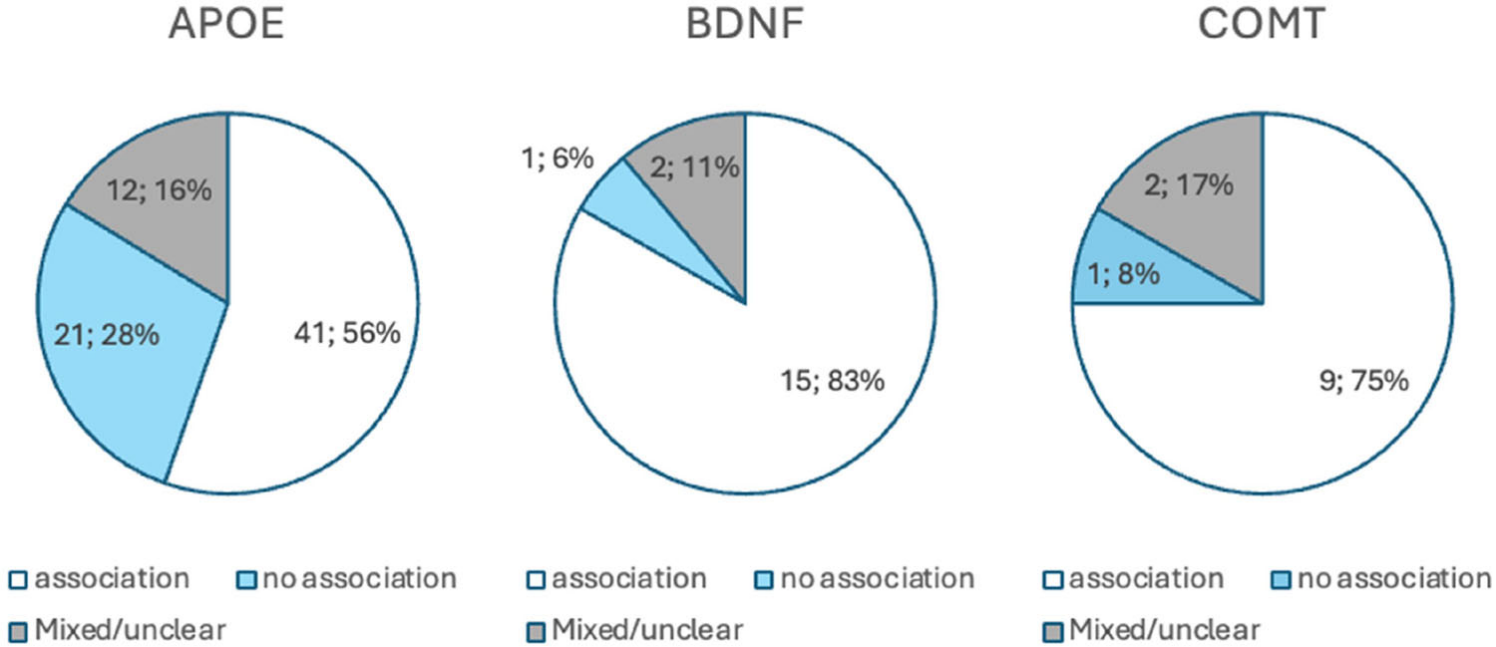
Association of *APOE*, *BDNF*, and *COMT* with cognitive outcomes.

Three genes were investigated in more than 10 studies each, and the findings are detailed in the following sections and summarized in Figure 2. Several other genes were only investigated by a few studies, highlighted in Supplementary Table 3.

Across the 121 included studies, 48 (40%) employed comprehensive neuropsychological test batteries, while 33 (27%) relied on more limited neuropsychological protocols. Brief cognitive screening tools were used in 28 studies (23%), and IQ assessments were the focus in six studies (5%). Only a small number of studies used dementia (*n* = 3) or Alzheimer's disease–specific (n = 3) diagnosis tools, such as the *Diagnostic and Statistical Manual of Mental Disorders* (DSM) (First 2013).

The most commonly used individual assessments were the Mini‐Mental State Examination (Arevalo‐Rodriguez et al. 2015), applied in 31 studies, followed by verbal fluency tests (*n* = 27) (Henderson, Peterson, Patterson, Lambon Ralph, and Rowe 2023), the California Verbal Learning Test (CVLT) (*n* = 24) (Persinger et al. 2018), the Trail Making Test (TMT) (*n* = 23) (Llinàs‐Reglà et al. 2017), and Digit Span (*n* = 20) (Weitzner et al. 2022). Subtests or full versions of Wechsler scales, including the WAIS (Hartman 2009) and WMS (Prigatano 1978), were used in 17 and 10 studies, respectively. Among screening tools, the Montreal Cognitive Assessment (*n* = 10) (Aiello et al. 2022) and CAMCOG (*n* = 5) (Aprahamian et al. 2011) were notable. Other frequently used instruments included the Stroop test (*n* = 12) (Periáñez et al. 2021), the Rey–Osterrieth Complex Figure Test (*n* = 10) (Zhang et al. 2021), and the Wisconsin Card Sorting Test (*n* = 6) (Nyhus and Barceló 2009).

### 3.2 Findings for Most‐studied Genes (*APOE*, *BDNF*, *COMT*)

#### 3.2.1 APOE

The *Apolipoprotein E* gene (*APOE*) encodes apolipoprotein E, crucial for lipid transport and neuronal repair. Its ε4 allele is widely studied, often linked to cognitive decline and vulnerability to neurological disorders.

Of the studies included in this review, *APOE* was investigated in 34 of 57 (60%) of all TBI studies, 34 out of 46 74% of stroke studies, and 6 of 19 32% of studies on other injuries.

**FIGURE 3:**
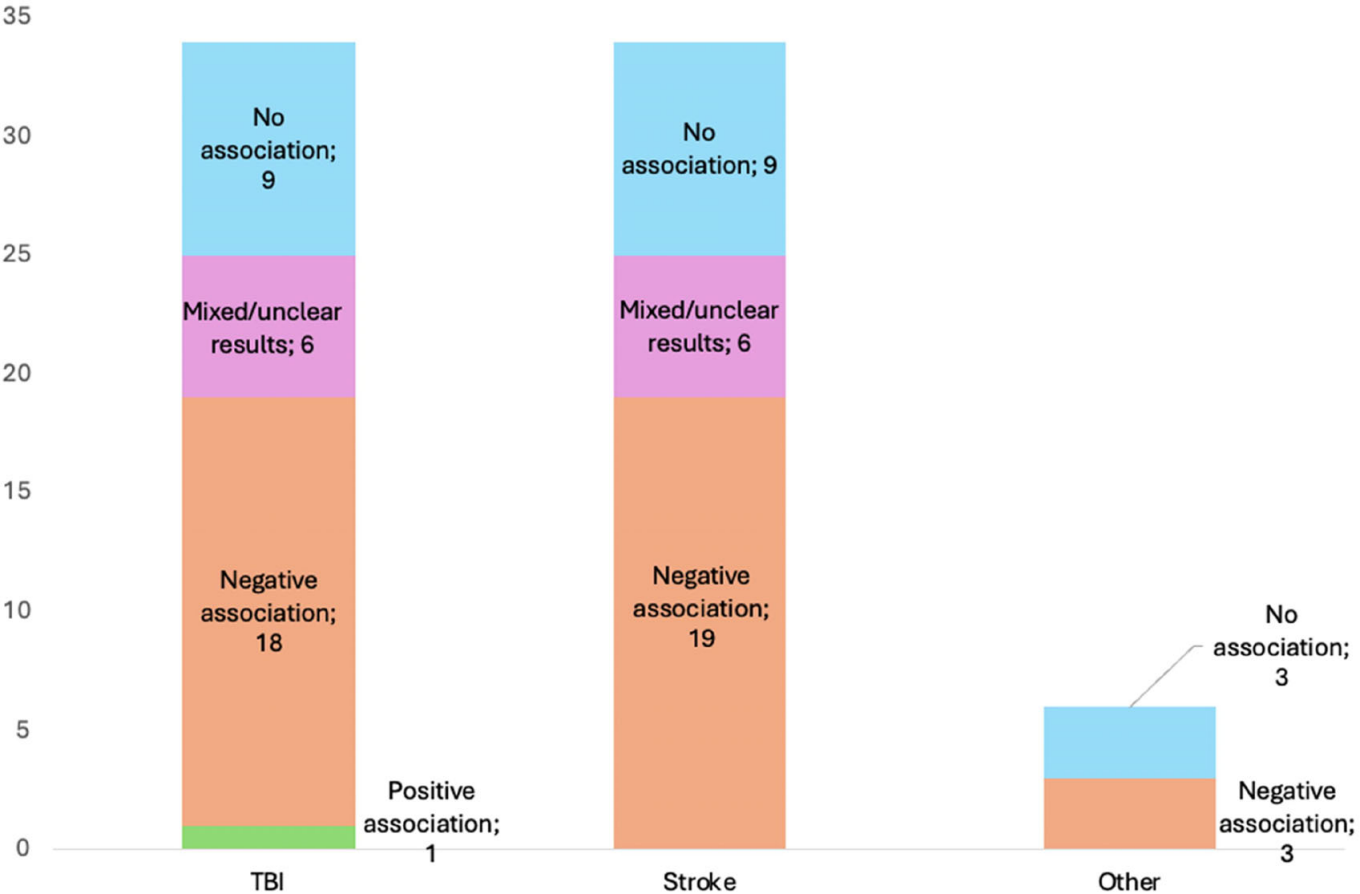
Association between *APOE* and cognitive outcomes for each brain injury type.

Forty‐one out of the total 74 *APOE* studies (55%) identified an association with cognitive outcomes, of which only one reported improved cognition in *APOE* ε4 carriers approximately one month post‐injury (S. D. Han et al. 2007). Among 40 studies with worsening cognitive outcomes, 29 (72%) were classified as low‐biased papers (Anderson et al. 2009; Ariza et al. 2006; Ballard et al. 2004; Baum et al. 2007; Correa et al. 2014, 2019; Crawford et al. 2002; Dik et al. 2000; Eramudugolla et al. 2014; Y. Han et al. 2020; Hellstrøm et al. 2022; Isoniemi et al. 2006; Johnson 2020; Johnston et al. 2000; Keins et al. 2021; Koponen et al. 2004; Lanterna et al. 2005; Liberman, Stewart, Wesnes, and Troncoso 2002; Llewellyn et al. 2010; Mauri et al. 2006; Merritt et al. 2018; Müller et al. 2009; Pendlebury et al. 2020; Percy et al. 2014; Shaaban et al. 2019; Tang et al. 1996; Wagle et al. 2009, 2010; Wooten et al. 2021), all of which reported impairments associated with APOE. While most of these focused on ε4 carriers, some studies referred to APOE more generally or compared allele distributions without isolating ε4 effects. No studies reported consistent positive associations with any APOE genotype. (Figure 3).

Furthermore, 51 of 74 (69%) studies specified their findings by investigating the ε4 allele or genotype combinations. In 36 of these 51 studies (71%) an association between ε4 and cognition was demonstrated, while 8 of 51 studies (16%) reported no associations between genotypes or alleles. When only considering low‐biased studies, 26 out of 36 (72%) found a negative association between ε4 allele and cognition.

The *APOE‐*ε4 allele was frequently associated with worse cognitive outcomes after stroke, particularly in memory and global cognition (Ballard et al. 2004; Reitz et al. 2006; Rowan et al. 2005; Werden et al. 2019). Studies by (Dik et al. 2000) and (Pendlebury et al. 2020) reported higher risk for dementia and cognitive decline among ε4 carriers, while (Lanterna et al. 2005) and (Werden et al. 2019) highlighted delayed recovery and structural brain changes. However, mixed or limited effects were noted in other studies (Qian et al. 2012; Sachdev et al. 2014), suggesting ε4's impact varies with factors like stroke severity and comorbidities.

Han et al. (2007) (S. D. Han et al. 2007) found that in a population with mild to moderate TBI, ε4‐carriers performed better than non‐carriers in several neuropsychological tests, both before and after controlling for injury severity, highlighting the mixed nature of the findings.

**FIGURE 4:**
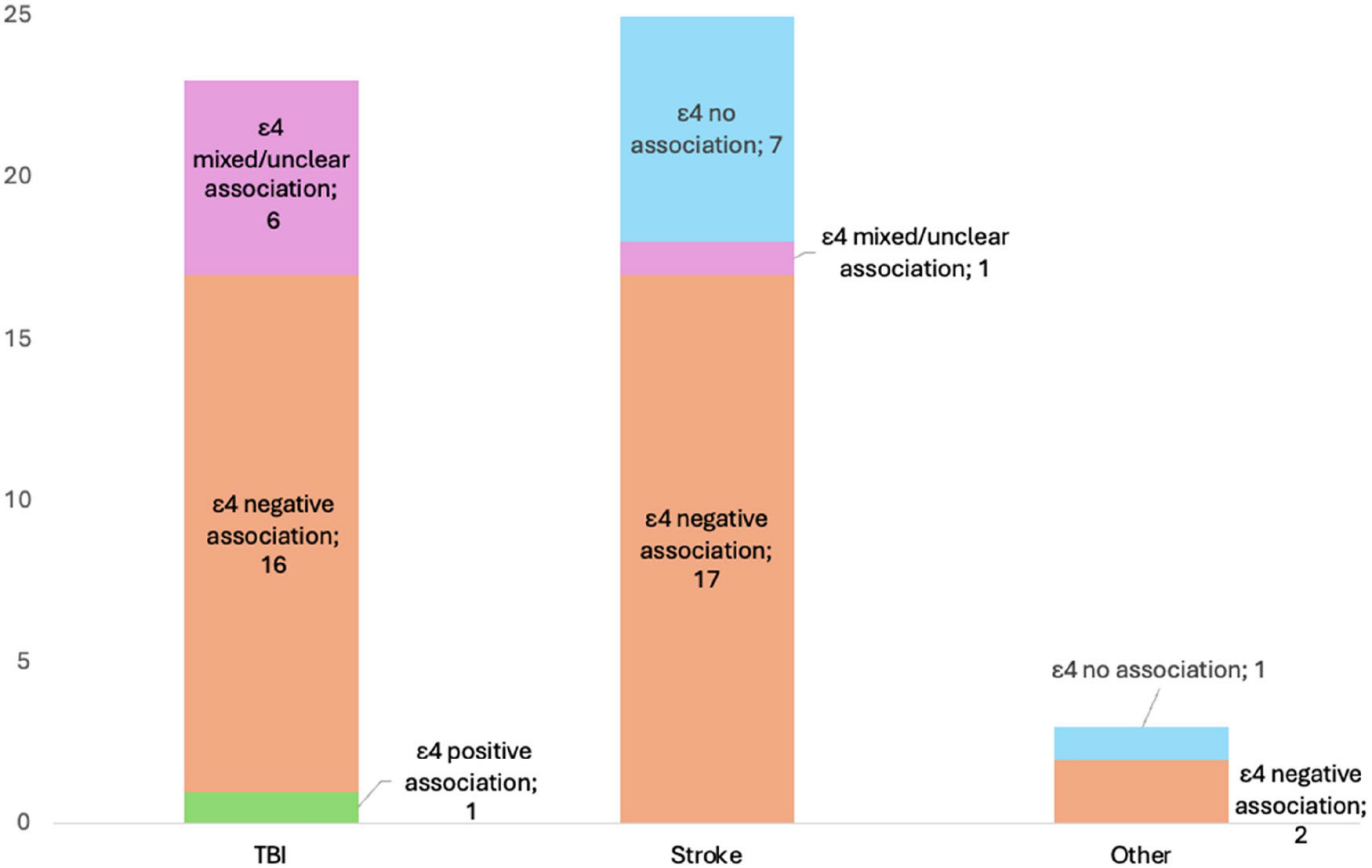
Association between the *APOE‐*ε4 allele and cognitive outcomes for each brain injury type.

However, most studies investigating mild to moderate TBI reported associations between ε4 carriage and cognitive impairment (Figure 4) (19, 35, 8, 40, 44, 45).

#### 3.2.2 BDNF

The *Brain‐derived neurotrophic factor* gene (BDNF) plays a critical role in neuroplasticity and dopamine pathways, making it a key focus in studies exploring genetic influences on cognitive recovery after brain injuries. The rs6265 polymorphism (Val66Met) is the most studied variant.

**FIGURE 5:**
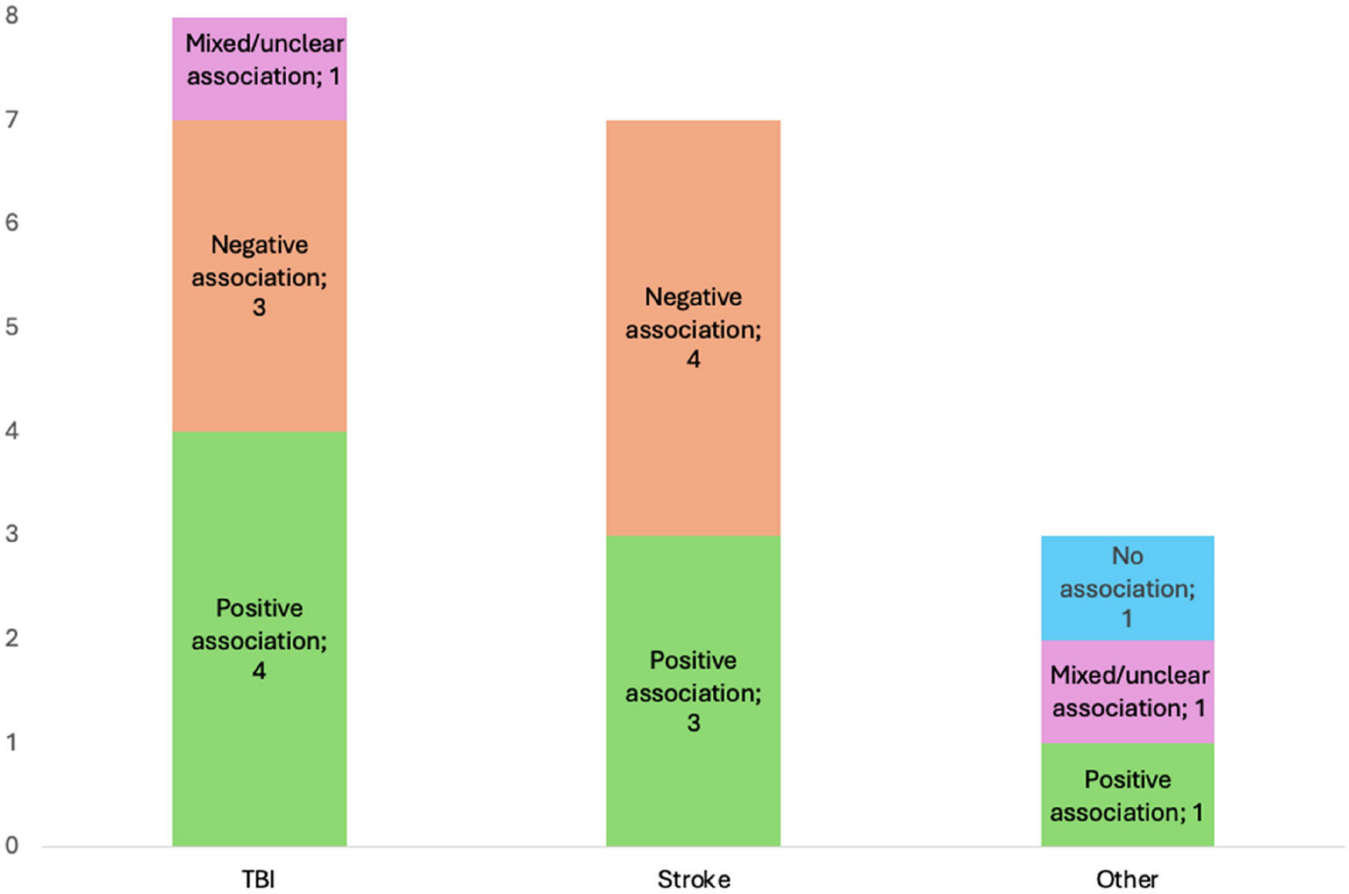
Association between *BDNF* rs6265 and cognitive outcomes for each brain injury.

**FIGURE 6:**
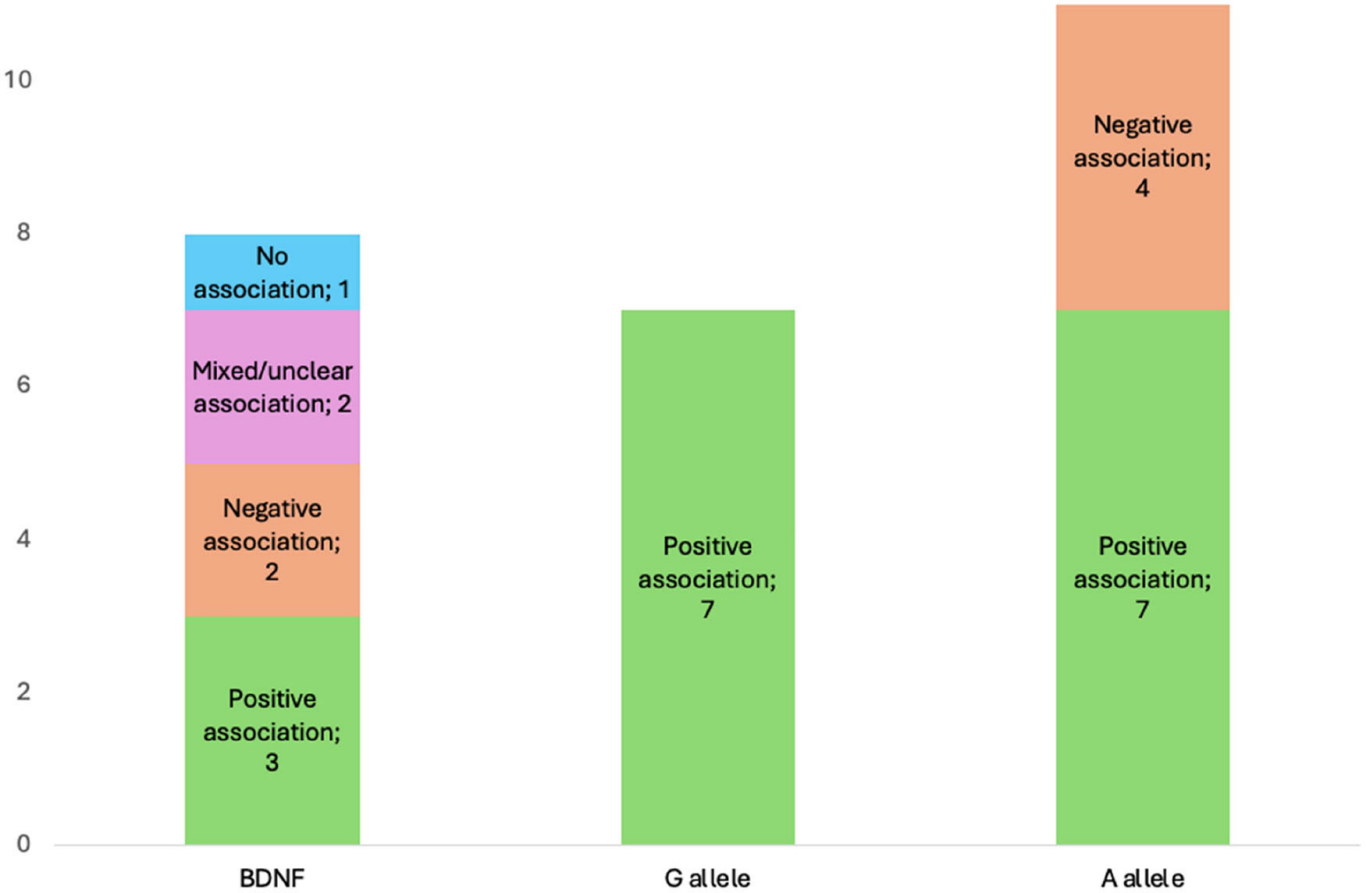
Association between *BDNF*, G allele, and A allele of the SNP rs6265 and cognitive outcomes.

All 18 studies examining *BDNF* focused on the rs6265 SNP. Of these, 15 reported a significant association with cognitive impairment, though the direction of the effect—whether positive or negative—was inconsistent. Seven suggested a protective effect of the G allele (Val variant) (Altshuler et al. 2019; Barbey et al. 2014; Kim et al. 2012; McAllister et al. 2012; Narayanan et al. 2016; Treble‐Barna et al. 2023; Vilkki et al. 2008) while seven suggested the same for the A allele (Met variant) (Keshavarz, Saberi, Sharafshah, Asgari, and Rezaei 2016; Krueger et al. 2011; Merritt et al. 2020; Rezaei, Asgari Mobarake, and Saberi 2022; S. Rezaei et al. 2016; Sajjad Rezaei et al. 2017; S. Rezaei et al. 2020). Twelve out of the 18 studies were rated as having a low risk of bias, including 10 that demonstrated an effect of *BDNF* on cognition post‐injury. Details are shown in Figures 5 and 6.

Three studies also looked at other *BDNF* SNPs in addition to rs6265. Correa et al. (2016) (Correa et al. 2016) analyzed 11 *BDNF* SNPs in brain tumor patients and found significant associations with cognitive performance for four SNPs, but no effects for rs6265. The SNPs rs10767664, rs10835210, rs11030104, and rs2030324 were linked to worse performance in executive functions and memory.

#### 3.2.3 COMT

The *Catechol‐O‐methyltransferase* gene (*COMT*), particularly its rs4680 polymorphism (Val158Met), has been widely studied for its role in cognitive processes like memory and executive function.

Of the 12 studies analyzed, nine reported worsened cognitive performance associated with the *COMT* gene. Eleven of these studies were rated as having a low risk of bias, including eight that demonstrated a significant effect of *COMT* on cognition post‐injury.

A consistent pattern emerged, with the functionally relevant G (Val) allele of rs4680, which increases *COMT* activity and lowers dopamine levels, being associated with worse cognitive outcomes. Correspondingly, several studies reported a protective effect of the A (Met) allele across various brain injuries (Altshuler et al. 2019; Correa et al. 2016; Correa et al. 2019; Kurowski et al. 2016; Lipsky et al. 2005; Liu et al. 2015; Nekrosius et al. 2019; Winkler et al. 2016).

**FIGURE 7:**
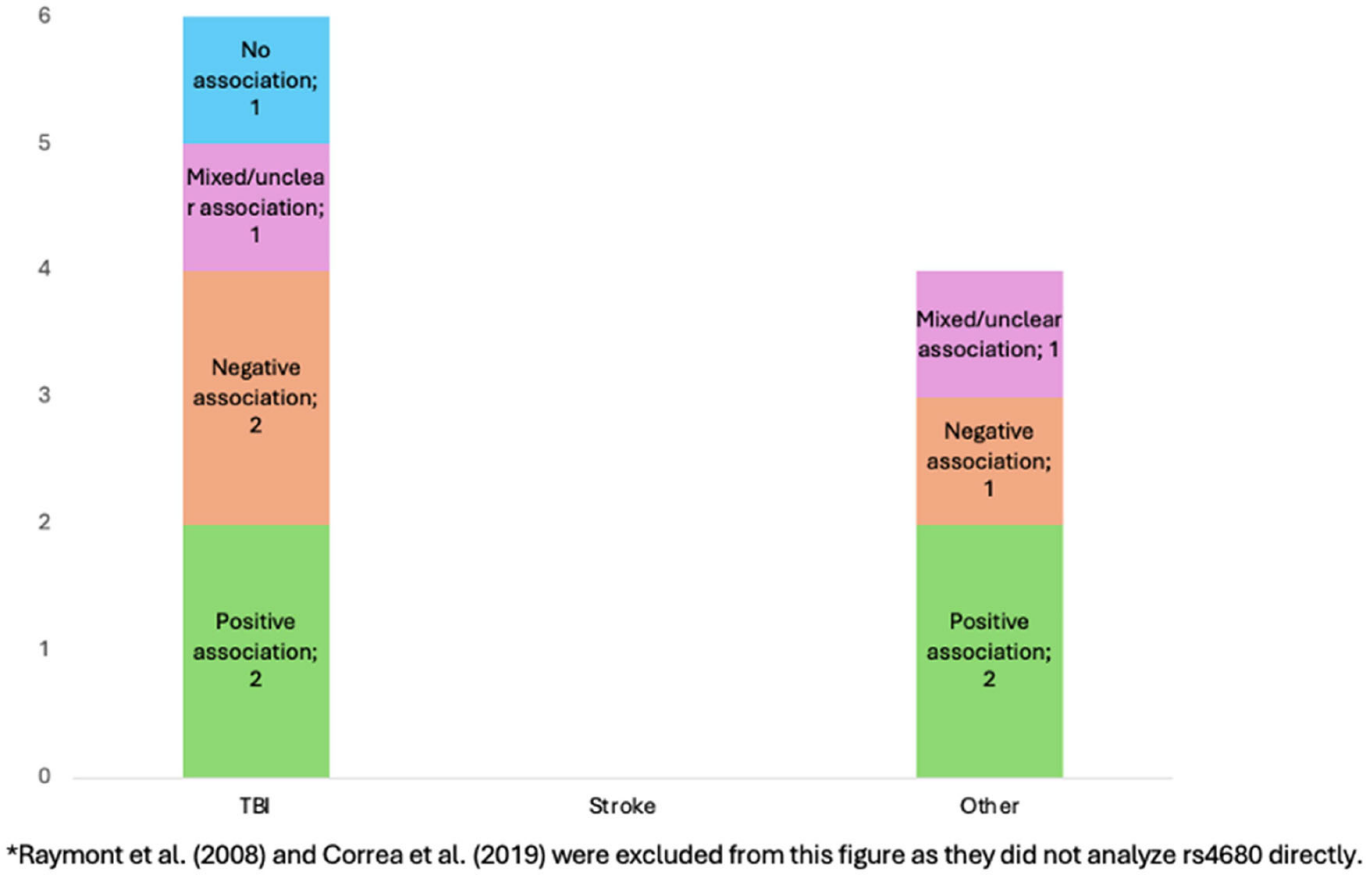
Association of *COMT* rs4680, with cognitive outcomes for each brain injury*.

**FIGURE 8:**
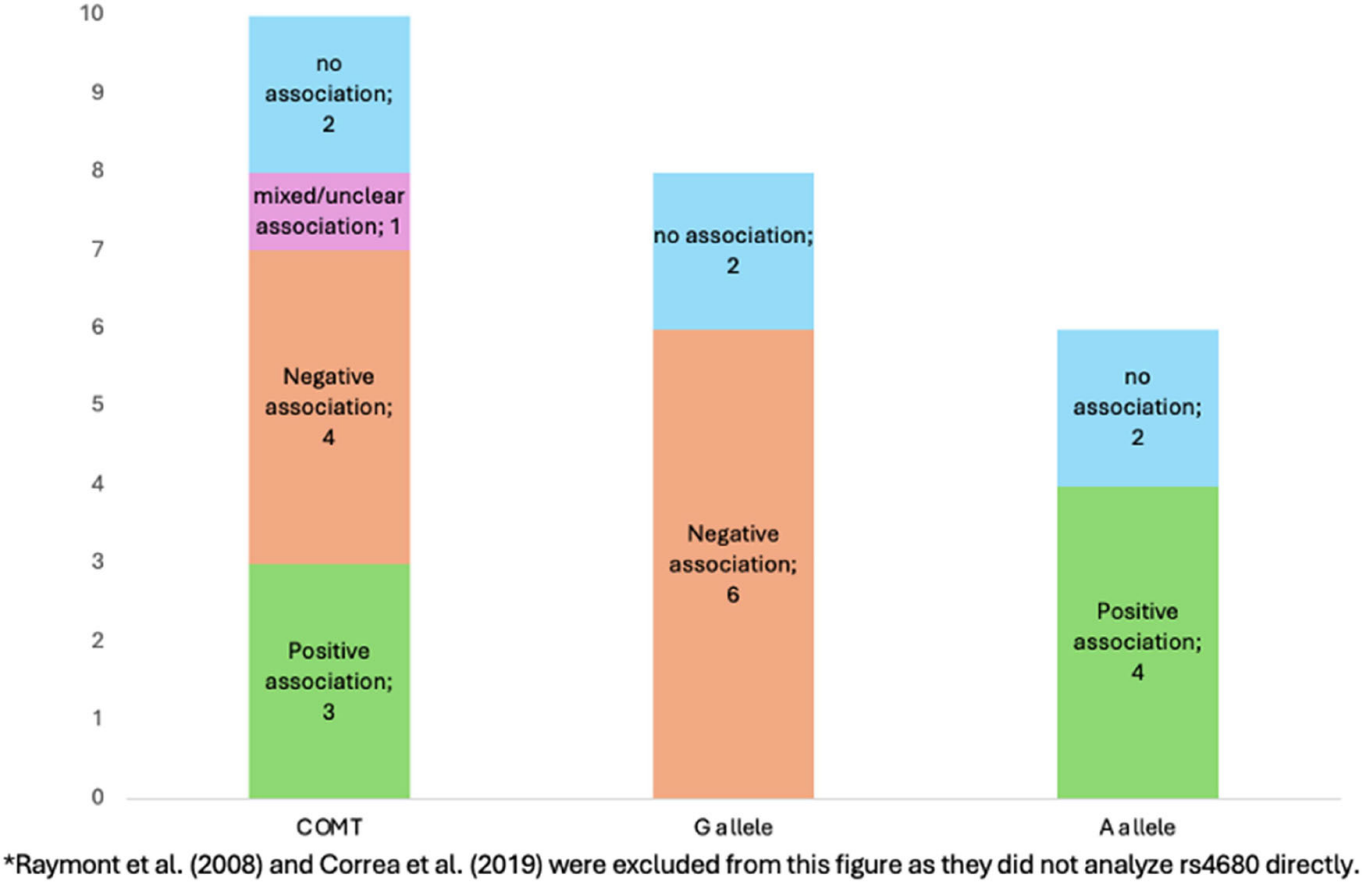
Association of *COMT* G allele, and A allele of the SNP rs4680 with cognitive outcomes*.

Howarth et al. (2014) (Howarth et al. 2014) compared genotypes in rs4680 and found that childhood brain tumor survivors with the A/G genotype performed better on computerized working memory tasks than those with the A/A genotype, with a significant difference observed only between these two groups. Details are displayed in Figures 7 and 8.

Correa et al. (2016) reported a significant association between *COMT* rs4680 (Val158Met) and delayed recall, with Val/Val (GG) carriers performing worse than Met/Met (AA) carriers. They also found that carriers of the minor allele—for example, AG or GG genotypes—for rs9332377, rs165815, rs4646312, rs5993883, rs4646316, and rs16815 had lower scores on tests of attention, executive function, and memory compared to major allele homozygotes. In contrast, minor allele carriers of rs4818, rs5746847, and rs6269 showed better cognitive performance (Correa et al. 2016).

Two studies did not analyze rs4680 but focused on other SNPs. Raymont et al. (2008) found that *COMT* rs9332330 significantly predicted cognitive recovery, with homozygotes showing better AFQT improvement than heterozygotes, while rs2020917 was associated with lower MMSE scores, though the direction of effect was not specified (Raymont et al. 2008). Correa et al. (2019) identified significant associations between cognitive impairment and four *COMT* SNPs—rs165815, rs9332377, rs174696, and rs740603—primarily affecting attention, executive function, and memory, though without specifying allele effects (Correa et al. 2019).

## 4 Discussion

The *APOE* ε4 allele was generally associated with worse cognitive outcomes in patients suffering from brain insults, with most studies reporting significant effects, though some reported no association. A similar pattern is observed for the G allele of the *COMT* rs4680 gene. The results for the *BDNF* gene, however, are more variable. The findings in this systematic review reflect the inconsistencies and challenges in interpreting genetic influences on cognitive recovery and outcomes across different conditions.

The *APOE* ε4 allele is present in approximately 8%–16% of the European population with notable regional variation. Population‐based studies have shown that its frequency declines with age, likely reflecting increased mortality among carriers (McKay et al. 2011).

While a majority of studies support the association of *APOE* polymorphisms with cognitive impairment across a range of brain insults (Alfieri et al. 2008; Anderson et al. 2009; Ariza et al. 2006; Ballard et al. 2004; Correa et al. 2018; Correa et al. 2014; Crawford et al. 2002; Dik et al. 2000; Eramudugolla et al. 2014; Y. Han et al. 2020; Hellstrøm et al. 2022; Isoniemi et al. 2006; Johnson 2020; Johnston et al. 2000; Keins et al. 2021; Koponen et al. 2004; Lanterna et al. 2005; Liberman et al. 2002; Llewellyn et al. 2010; Mauri et al. 2006; Merritt et al. 2018; Jaleel et al. 2021; Müller et al. 2009; Pendlebury et al. 2020; Percy et al. 2014; Qian et al. 2012; Rippon et al. 2006; Sachdev et al. 2014; Shaaban et al. 2019; Sundström et al. 2004; Sundström et al. 2007; Tang et al. 1996; Teasdale, Jorgensen, Ripa, Nielsen, and Christensen 2000; Veeramuthu et al. 2014; Wagle et al. 2009, 2010; Werden et al. 2019; Wooten et al. 2021; Yue et al. 2017), some literature on TBI (Nathoo et al. 2003) and ischemic stroke (Khan et al. 2013) have reported no association or mixed results with carrier‐status. In relation to brain tumors, the role of the *APOE*‐ε4 allele is less studied, with either no evidence or negative outcomes on cognition (Butterbrod et al. 2021; Correa et al. 2018; Correa et al. 2014, 2019; Huntoon et al. 2023). In contrast, the study by Correa et al. (2014) (Correa et al. 2014) did find an increased susceptibility for the ε4 allele and cognitive deficits in brain tumor patients. The observed pattern in this systematic review is supported by previous studies (Gharbi‐Meliani et al. 2021; Xu, Liang, and Huang 2024), which also report an association between APOE‐ε4 and worsening cognitive trajectories after brain insults. However, the complexity of *APOE*‐related pathways and variability between diagnoses (Dilliott et al. 2021; Lyall et al. 2016) highlight the need for further targeted research.

This review further highlights the inconsistent findings of the *BDNF* gene across the studies included in this systematic review. Beyond its role in Alzheimer's disease, the *BDNF* gene and its encoded protein have been implicated in numerous other neurological and cardiovascular diseases. A recent study by Koyya et al. (2024) suggests that the BDNF protein acts as a protective factor against Alzheimer's disease, Parkinson's disease, Huntington's disease, and cardiovascular diseases (Kaess et al. 2015). This heterogeneity in neurological diseases exemplifies the complex mechanisms by which *BDNF* influences cognition. A review by Angoa‐Pérez et al. (2017) highlighted the dual roles of *BDNF*, which exists as a precursor (pro‐BDNF) and mature form (m‐BDNF). These forms bind to different receptors—p75NTR and TrkB—mediating opposing effects that may help explain inconsistent findings across studies. TrkB promotes neuronal survival and plasticity, while p75NTR can induce trophism or apoptosis depending on the context. These opposing mechanisms likely contribute to inconsistencies across studies.

In conditions like Alzheimer's disease (Boots et al. 2017; Franzmeier et al. 2021; Lim et al. 2021) and schizophrenia (Farcas, Hindmarch, and Iftene 2023; Schweiger et al. 2019; Ursini et al. 2016), the rs6265 (Val66Met) polymorphism could often be associated with a negative effect on cognition, with the Met (A) allele linked to reduced *BDNF* production and cognitive decline. However, these findings may not generalize to brain injuries like TBI or stroke, where *BDNF*’s role varies by injury‐specific and molecular contexts.

This systematic review reinforces that the G allele of *COMT* rs4680 (Val allele) is consistently linked to worse cognitive outcomes, likely due to its role in increasing *COMT* activity and lowering dopamine levels. This supports prior research indicating its involvement in cognitive dysfunction across various neurological conditions, including schizophrenia (Huang et al. 2016), and suggests a potential target for intervention. In addition, prior research suggests that the A allele of *COMT* rs4680 may act as a protective factor of cognition in various neurological conditions and aging populations (Cha et al. 2022).

The majority of included studies, such as Winkler et al. (2016) (Winkler et al. 2016), align with this notion, showing improved cognitive outcomes in A/A carriers. However, a few others (e.g., Brown et al. 2023; Willmott et al. 2014) reported no significant effects. These discrepancies make it difficult to reliably attribute a protective role of the A allele of rs4680 across different brain injuries and conditions.

Lanni et al. (2012) proposed a synergistic interaction between the *COMT* rs4680 G/G genotype and *APOE*‐ε4, increasing susceptibility to cognitive impairments. This interaction, however, was not explored in the studies included in our review, which focused instead on the independent effects of the *COMT* rs4680 polymorphism on cognition across brain injuries.

Investigating other genetic pathways, such as those related to inflammation, DNA repair, and metabolism, in conjunction with injury type and severity, could provide more comprehensive insights into the genetic underpinnings of cognitive outcomes.

This systematic review has some notable limitations. Data distribution was uneven, with *APOE*‐ε4 being well‐studied (74 studies), while other genes, like *ACE, GST*, and *MTHFR*, were investigated in fewer than 10 studies. Further, many studies lacked statistical power to find associations and were likewise prone to spurious findings. The focus on specific genotypes or allele carrier status in some studies may limit the generalizability of findings. There was also variability in cognitive assessment methods and follow‐up durations; 39% of longitudinal studies had follow‐ups ≤6 months, potentially limiting insights into long‐term outcomes.

Inconsistencies likely reflect heterogeneity in injury phenotype/severity and treatment, testing windows, small samples, heterogeneous cognitive measures, population structure, genotyping/quality control, and untested interactions. The following is a proposal to guide future research in the field:

First, adequately powered multicenter studies and clearer standardization of injury types would improve the comparability of findings and support more robust synthesis across acquired brain injuries. Second, greater consistency in the timing of cognitive assessments, together with the use of a core cognitive test battery, would facilitate meaningful comparison between studies. Third, genetic studies would benefit from transparent reporting of ancestry and the use of longitudinal analytical approaches that provide effect sizes and confidence intervals and explore potential interactions.

Finally, integrating imaging and other biomarkers may help clarify mechanistic pathways linking genetic factors to cognitive outcomes.

Despite these limitations, this systematic review offers key strengths. It provides a comprehensive overview of the most studied genetic markers across various brain injuries, populations, and demographics. The majority of studies were longitudinal in design, which enhances the understanding of cognitive outcomes over time, hence enabling more reliable inferences.

The heterogeneity of studies suggests that further research with more standardized protocols may be beneficial for future comparisons.

## 5 Conclusion

This systematic review examined genetic risk factors related to cognitive outcomes following brain insults. The most studied genes were *APOE*, *BDNF*, and *COMT*. Among these, *APOE*‐ε4 consistently emerged as a significant factor associated with poorer cognitive recovery, not only in dementia but also across various types of brain injuries. Additionally, multiple studies reported that the G allele of *COMT rs4680* is linked to worse cognitive outcomes. While these findings align with previous research, other genetic associations, including those related to *BDNF*, remain less consistent. Future research should focus on validating these genetic markers in larger datasets to further understand their role in post‐injury cognitive recovery.

## Author Contributions

**Tora Dunås**: conceptualization, methodology, data curation, and writing – original draft. **Sophia M. Leiss**: data curation, formal analysis, visualization, and writing – original draft. **Alba Corell**: writing – review and editing. **Thomas Skoglund**: writing – review and editing. **Anna Dénes**: writing – review and editing. **Helena Carén**: writing – review and editing. **Anja Smits**: writing – review and editing. **Isabelle Rydén**: writing – review and editing. **Asgeir S. Jakola**: conceptualization and writing – review and editing.

## Funding

The study was financed by grants from the Swedish state under the agreement between the Swedish government and the county councils, the ALF‐agreement: ALFGBG‐1006089.

## Conflicts of Interest

The authors declare no conflicts of interest.

## Supporting information




**Supplementary Materials**: brb371173‐sup‐0001‐SuppMat.docx

## Data Availability

This systematic review is based on data extracted from published literature. The extracted datasets and supporting materials are available from the corresponding author upon reasonable request.
