# Supplementary material for: Genetic Risk Factors for Poor Cognitive Outcome Following Brain Insult—A Systematic Review
Source: Brain Behav. 2025 Dec 31;16(1):e71173. doi: 10.1002/brb3.71173 (PMC12755970; doi:10.1002/brb3.71173)
Supplement: Supplementary file 1 — Supplementary Materials: brb371173‐sup‐0001‐SuppMat.docx [file BRB3-16-e71173-s001.docx]

**Supplementary 1: Search promt.**

((Polymorphism, Genetic[mesh] OR genes[mesh] OR genes[tiab] OR Genetic*[tiab] OR polymorphism*[tiab] OR *APOE*[tiab] OR *APOE*[mesh])

AND

(Cognition[mesh] OR cogniti*[tiab] OR neuropsychologic*[tw])

AND

(stroke[mesh] OR stroke[tiab] OR brain neoplasms[mesh] OR brain injuries[mesh] OR intracranial hemorrhages[mesh] OR brain hemorrhage*[tiab] OR brain tumo*[tiab] OR brain injur*[tiab] OR brain cancer*[tiab] OR neural injur*[tiab] OR glioma[mesh] OR glioma[tiab] OR brain surgery [tiab]))

NOT (((animals[mesh]) NOT (animals[mesh] AND humans[mesh])))

**Supplementary 2: Assessment of bias.**

| **Author** | **Year** | **Checklist** | **Bias risk** | **common limitations** |
| --- | --- | --- | --- | --- |
| Alfieri | 2008 | longitudinal | high | Small sample size (n=30) |
| Allan | 2011 | longitudinal | low |  |
| Altshuler | 2019 | longitudinal | low | Confounding not addressed; small sample size (n=42) |
| Anderson | 2009 | case control | low | Dropout effects on outcomes not addressed |
| Ariza | 2006 | longitudinal | low |  |
| Ariza | 2006 | longitudinal | low | Dropout at 12-month follow-up; effect on outcomes not explored |
| Ballard | 2004 | longitudinal | low |  |
| Barahmani | 2009 | longitudinal | medium | Small sample size (n=20) |
| Barba | 2000 | longitudinal | medium |  |
| Barbey | 2014 | longitudinal | low |  |
| Baum | 2007 | longitudinal | low |  |
| Bour | 2010 | longitudinal | low |  |
| Brown | 2023 | longitudinal | low |  |
| Butterbrod | 2021 | longitudinal | medium |  |
| Chamelian | 2004 | longitudinal | low |  |
| Correa | 2014 | cross-sectional | low | Small sample size (n=23) |
| Correa | 2016 | cross-sectional | low | Small sample size (n=49) |
| Correa | 2018 | longitudinal | high |  |
| Correa | 2019 | cross-sectional | low | Small sample size (n=18) |
| Crawford | 2002 | longitudinal | low | Small sample size (n=18) |
| Del Mar Matarín | 2005 | longitudinal | low |  |
| Dharmasaroja | 2020 | longitudinal | low | Lacks sample size calculation and power analysis |
| Dik | 2000 | longitudinal | low |  |
| Donnellan | 2019 | case control | low |  |
| Eramudugolla | 2014 | longitudinal | low |  |
| Failla | 2015 | longitudinal | low |  |
| Gong | 2021 | longitudinal | low | Only subset in follow-up (n=46) |
| Han | 2007 | case control | low |  |
| Han | 2020 | longitudinal | low | Small sample size (n=39 patients; 27 healthy subjects) |
| Hellstrøm | 2022 | longitudinal | low |  |
| Hodgkinson | 2009 | longitudinal | low |  |
| Howarth | 2014 | longitudinal | low |  |
| Huntoon | 2023 | longitudinal | low | 35% loss to follow-up |
| Ismail | 2021 | longitudinal | low |  |
| Isoniemi | 2006 | longitudinal | low | Missing data handling and dropout details not reported |
| Jaleel | 2021 | longitudinal | high |  |
| Johnson | 2020 | longitudinal | low | Dropouts due to declining genetic screening (n=48) |
| Johnston | 2000 | case control | low |  |
| Mary T. Joy | 2019 | longitudinal | low | Small sample size (n=44) |
| Kautiainen | 2020 | cross-sectional | high |  |
| Kautiainen | 2022 | cross-sectional | high | Sample size limited (n=81), but robust methodology |
| Keins | 2021 | longitudinal | low |  |
| Keshavarz | 2016 | case control | low | No pre-injury cognitive data |
| Kim | 2012 | longitudinal | low | Potential baseline cognitive impairment |
| Klimkowicz | 2005 | cross-sectional | medium | Follow-up data lacking |
| Knopman | 2009 | cross-sectional | low |  |
| Koponen | 2004 | cross-sectional | low | No matching; unclear post-stroke cognitive timing |
| Krueger | 2011 | cross-sectional | low | Confounding not addressed |
| Kurowski | 2016 | longitudinal | low | Confounding not addressed |
| Lanterna | 2005 | longitudinal | low | No pre-stroke cognition data; dropout and FU not discussed |
| Liberman | 2002 | longitudinal | low |  |
| Lipsky | 2005 | longitudinal | low | Small sample size (n=19) |
| Liu | 2015 | cross-sectional | low | No matching; adjusted for group differences |
| Llewellyn | 2010 | cross-sectional | low | Follow-up description lacking (n=34) |
| Louko | 2006 | longitudinal | medium | Follow-up description lacking |
| Mangone | 2020 | longitudinal | low |  |
| Markos | 2017 | longitudinal | low | Small sample size (n=39) |
| Mauri | 2006 | longitudinal | low |  |
| McAllister | 2005 | longitudinal | high | Only 21 patients, 3 in risk group |
| McAllister | 2008 | cross-sectional | low |  |
| McAllister | 2012 | case control | low | 32% loss to follow-up |
| Merritt | 2018 | cross-sectional | low |  |
| Merritt | 2018 | cross-sectional | low | Post hoc analysis; small sample size (n=40) |
| Merritt | 2020 | cross-sectional | low | Small groups per gene (n=32); baseline data only |
| Merritt | 2021 | cross-sectional | low |  |
| Millar | 2003 | longitudinal | medium | Unclear genotyping method; dropout info missing |
| Mok | 2012 | longitudinal | low |  |
| Moran | 2009 | longitudinal | low | Mixed studies; one drug trial with no effect |
| Morris | 2004 | longitudinal | medium | Longitudinal design; cognition only assessed at 6 months |
| Morris | 2011 | longitudinal | medium |  |
| Müller | 2009 | longitudinal | low | No individual matching |
| Narayanan | 2016 | longitudinal | medium | Group comparison lacks individual-level matching |
| Nekrosius | 2019 | longitudinal | low | Small sample size (n=30) |
| Noé | 2010 | longitudinal | low |  |
| Oyefiade | 2019 | longitudinal | medium | Confounding not addressed; small sample size (n=42) |
| Padgett | 2016 | longitudinal | low | Dropout effects on outcomes not addressed |
| Pendlebury | 2020 | longitudinal | low |  |
| Percy | 2014 | case control | low | Dropout at 12-month follow-up; effect on outcomes not explored |
| Plassman | 2000 | longitudinal | low |  |
| Pruthi | 2010 | longitudinal | medium | Small sample size (n=20) |
| Qian | 2012 | longitudinal | low |  |
| Rajan | 2016 | longitudinal | low |  |
| Rapoport | 2008 | case control | low |  |
| Raymont | 2008 | longitudinal | medium |  |
| Reitz | 2006 | longitudinal | medium |  |
| Rezaei | 2016 | longitudinal | low |  |
| Rezaei | 2017 | case control | low |  |
| Rezaei | 2020 | case control | low | Small sample size (n=23) |
| Rezaei | 2022 | case control | high | Small sample size (n=49) |
| Rippon | 2006 | longitudinal | medium |  |
| Rostami | 2011 | longitudinal | medium | Small sample size (n=18) |
| Rowan | 2005 | longitudinal | medium | Small sample size (n=18) |
| Sachdev | 2014 | longitudinal | medium |  |
| Shaaban | 2019 | longitudinal | low | Lacks sample size calculation and power analysis |
| Shadli | 2011 | case control | high |  |
| Shee | 2016 | case control | medium |  |
| Sundström | 2004 | longitudinal | high |  |
| Sundström | 2007 | longitudinal | medium |  |
| Tang | 1996 | case control | low | Only subset in follow-up (n=46) |
| Teasdale | 2000 | longitudinal | high |  |
| Treble | 2023 | longitudinal | low | Small sample size (n=39 patients; 27 healthy subjects) |
| Tripodis | 2017 | case control | low |  |
| Veeramuthu | 2014 | longitudinal | high |  |
| Vilkki | 2008 | longitudinal | low |  |
| Wagle | 2009 | cross-sectional | low | 35% loss to follow-up |
| Wagle | 2010 | longitudinal | low |  |
| Wagner | 2012 | longitudinal | low | Missing data handling and dropout details not reported |
| Wefel | 2022 | longitudinal | medium |  |
| Werden | 2019 | case control | high | Dropouts due to declining genetic screening (n=48) |
| Willmott | 2013 | longitudinal | high |  |
| Willmott | 2014 | longitudinal | medium | Small sample size (n=44) |
| Winkler | 2016 | cross-sectional | low |  |
| Wooten | 2021 | cross-sectional | low | Sample size limited (n=81), but robust methodology |
| Yang | 2022 | longitudinal | high |  |
| Yang | 2022 | longitudinal | medium | No pre-injury cognitive data |
| Yu | 2021 | cross-sectional | low | Potential baseline cognitive impairment |
| Yue | 2015 | cross-sectional | medium | Follow-up data lacking |
| Yue | 2017 | cross-sectional | medium |  |
| Yue | 2017 | cross-sectional | medium | No matching; unclear post-stroke cognitive timing |
| Zeng | 2019 | case control | high | Confounding not addressed |
| Zhao | 2022 | case control | medium | Confounding not addressed |

**Supplementary 3: Pronounced results for each gene.**

| **Gene/SNP** | **No. of articles, n (%)** | **Total no. of patients** | **Main outcomes** | **Proposed association with congitive outcomes** |
| --- | --- | --- | --- | --- |
| ***APOE*** | 74 (61%) | 34.043 | *E4*: deficit on general cognitive ability.  ***rs584007***: positive on verbal fluency | *E4*: typically negative effects (outside of TBI research)  *rs584007*: positive effects |
| ***BDNF*** | 18 (15%) | 3.458 | ***rs11030104*** and  ***rs10767664***: impairments in **memory** and **learning tasks** | *rs6265*: most reported but inconsistant.  ***rs11030104*** and ***rs10767664*: impairments in brain tumor and TBI** |
| ***COMT*** | 12 (10%) | 2.241 | **A allele of *rs4680***: protective for Memory and executive tasks  G allele/ other SNPs:  Deficits in memory, learning, attention | The **A allele of *rs4680***: protective  **G allele** and specific SNPs (e.g., *rs16815*) are associated with deficits. |
| ***DRD1/DRD2/ANKK1*** | 8 (7%) | 1.138 | E.g., *rs6277 (C957T)* and *rs6279*: linked to better outcomes in verbal memory and general cognitive performance.  E.g., *rs34863235*: linked to worse memory performance. | ***ANKK1 Taq1A (rs1800497)*** and ***DRD2 rs6277*** are the most studied SNPs.  T allele: inconsistant findings. |
| ***GSTM1/T1/P1*** | 5 (4%) | 495 | ***GSTM1* and *GSTT1*: null genotypes** consistently show negative effects on cognitive outcomes, particularly post-treatment. | ***GSTP1*** is the most investigated gene, but findings on SNPs like ***rs1695*** and ***rs1138272*** vary between studies |
| ***ACE*** | 4 (3%) | 630 | DD genotype: poorer outcomes in memory, executive function, attention, and processing speed.  I/I genotype: protective for memory | The **D allele** (especially the **DD genotype**) is generally associated with **poorer cognitive outcomes**.  The **I/I genotype** appears protective or associated with better cognitive outcomes |
| ***MTHFR*** | 3 (3%) | 322 | *rs1801133*/ *rs1801131*: greater verbal and working memory decline | *rs1801133*/ *rs1801131***:**  negative |
| ***PPAR*** | 3 (3%) | 518 | FSIQ – general cognitive ability | ***rs6008197* C allele: protective**  *rs6008197*: negative |
| ***DRD1/DRD2/ANKK1*** | 8 (7%) | 1.138 | E.g., *rs6277 (C957T)* and *rs6279*: linked to better outcomes in verbal memory and general cognitive performance.  E.g., *rs34863235*: linked to worse memory performance. | ***ANKK1 Taq1A (rs1800497)*** and ***DRD2 rs6277*** are the most studied SNPs.  T allele: inconsistant findings. |
| ***GSTM1/T1/P1*** | 5 (4%) | 495 | ***GSTM1* and *GSTT1*: null genotypes** consistently show negative effects on cognitive outcomes, particularly post-treatment. | ***GSTP1*** is the most investigated gene, but findings on SNPs like ***rs1695*** and ***rs1138272*** vary between studies |
| ***ACE*** | 4 (3%) | 630 | DD genotype: poorer outcomes in memory, executive function, attention, and processing speed.  I/I genotype: protective for memory | The **D allele** (especially the **DD genotype**) is generally associated with **poorer cognitive outcomes**.  The **I/I genotype** appears protective or associated with better cognitive outcomes |
| ***MTHFR*** | 3 (3%) | 322 | *rs1801133*/ *rs1801131*: greater verbal and working memory decline | *rs1801133*/ *rs1801131***:**  negative |
| ***PPAR*** | 3 (3%) | 518 | FSIQ – general cognitive ability | ***rs6008197* C allele: protective**  *rs1800206*: positive  *rs6008197*: negative |
| ***GCLM*** | 1 | 121 | General cognition | negative |
| ***DTNBP1*** | 2 | 150 | *rs6008197* and *rs2619522* in brain tumors and their effect on memory | Negative on memory |
| ***ERCC2*** | 1 | 150 | *rs13181*  in brain tumors | Negative on general cognition |
| ***XRCC1*** | 1 | 150 | rs25487  in brain tumors | Mixed results |
| ***CCR*** | 2 | Ca. 279 | *CCR5-D32* mutation, *CCR2*: *rs7853346*, *rs1799864* | *CCR5*: positive in stroke and TBI recovery,  Mixed results for *CCR2*:  *rs7853346* CG/GG genotype protective; Negative: *rs1799864* |
| ***ACT*** | 1 | 142 | TT genotype in stroke | Negative association with TT genotype |
| ***GBA*** | 1 | 208 | General cognition in Parkinson’s | negative |
| ***VMAT2*** | 1 | 136 | rs363226 in severe TBI | negative |
| ***NOS3*** | 1 | 253 | TT genotype in stroke | TT genotype: negative |
| ***HFE*** | 1 | - | **H63D variant in stroke** | No significant effect |
| ***GRIN*** | 1 | 97 | In TBI | No significant effect |
| ***DBH*** | 1 | 97 | In TBI | negative |
| ***GAD*** | 1 | 97 | In TBI | Mixed results |
| ***SNCA*** | 1 | 91 | *rs1372525* in mild TBI | Positive on memory |
| ***KIBRA*** | 1 | 129 | *rs17070145* in severe | T allele: negative on episodic memory |
| ***PTENP1*** | 1 | 279 | In glioma patients | No effect |
| ***CCR2*** | 1 | 279 | *rs7853346*, *rs1799864* in glioma patients | Mixed:  *rs7853346* CG/GG genotype protective; Negative: *rs1799864* |
| ***ALDH2*** | 1 | 183 | *rs671* in stroke | negative |
| ***CST3*** | 1 | 152 | B allele in stroke | Negative for B allele: impaired vascular cognitive impairment |
| ***MMP-9*** | 1 | 148 | *rs3918242* in stroke | Negative: for TC/CC genotype |
